# Editorial: Insights in endocrinology of aging: 2022

**DOI:** 10.3389/fendo.2023.1228362

**Published:** 2023-06-08

**Authors:** James M. Harper

**Affiliations:** Department of Biological Sciences, Sam Houston State University, Huntsville, TX, United States

**Keywords:** type 2 diabetes mellitus, anti-diabetics, oral health, ovarian aging, menopause, cognition

As the world’s population grows older, the prevalence of age-related pathologies within the populace has steadily increased. Of these, Type 2 Diabetes Mellitus (T2DM) has become a major challenge facing healthcare systems worldwide. Moreover, as the collective age of females in the population also increases, the maintenance of female reproductive health within the aging population is paramount. This Research Topic focuses on some of the functional outcomes associated with T2DM and its implications for the aging population in conjunction with the reproductive health of aged females.

Many of the pathophysiological consequences of T2DM, especially retinal and renal disease, are well-known and well-studied. Peripheral neuropathy is also prevalent in patients with T2DM, most often due to axonal degeneration. An elevated level of neurofilament (Nf) protein subunits in cerebral spinal fluid (CSF) and blood is a well-known marker of neurodegeneration. Unfortunately, Nf is typically undetectable in blood which made CSF sampling the only practical diagnostic approach used in the past. Recently, however, single molecule array (Simoa) immunoassay technology has made it possible to quantify trace amounts of individual biomarkers which was exploited by Thota et al. to ask whether there was an association between glycemic state (e.g., euglycemia versus hyperglycemia) and the level of the Nf light chain (NfL) chain in the blood individuals with poor glycemic control relative to those whose glycemic control was normal. Not unexpectedly, individuals with T2DM had a significantly elevated level of blood NfL, but so too did pre-diabetic individuals suggesting that poor glycemic control is a risk factor for a neurodegenerative condition, even in the absence of overt DM.

Although peripheral neuropathy is common with T2DM, little attention has been given to the oral health of diabetic individuals. In a study of community-dwelling individuals in Shanghai, Da et al. used the Eichner index to examine the relationship between T2DM and occlusal support. Diabetic status was determined using common markers of glycemic control (i.e., blood glucose and/or hemoglobin A1c level) and a direct relationship between degree of glycemic control and occlusal support was found. Specifically, individuals with impaired glycemic control scored poorly on the Eichner Index, while the risk of having T2DM was significantly lower in individuals with good occlusal support. Poor occlusal support results in improper mastication of foodstuffs and an increased risk of a nutritional imbalance.

Currently, there are hundreds-of-millions of individuals suffering from T2DM globally, and although many anti-diabetics drugs already exist, there is a need to develop new or better ones. There is also a need to identify drugs than can work in combination with others to increase the benefits. Recently, free fatty acid receptor (FFAR1) agonists have gained attention as a therapeutic target for T2DM including that described by Rady et al. Using a combination of intact animals and isolated pancreatic islets from multiple species, they showed a convincing positive effect of their test compound on islet cell function as indicated by a reduction in both serum glucose and insulin level and an improved glucose clearance in intact animals. In addition, they found that treating intact islets from diabetic individuals or islets maintained under conditions to induce glucose toxicity resulted in an enhanced secretion of both insulin and glucagon. Continued work on this, and other FFAR1 agonists, hold a lot of promise in the treatment of T2DM.

Meanwhile, female reproductive health and the impact of menopause on functional outcomes is becoming increasingly important in geriatric medicine as the proportion of elderly females continues to increase. As noted by Wu et al. in their review article, the ovary is unique in that it ages at a more rapid pace than every other organ. While a precipitous decline in oocyte number and quality from puberty onset is the norm, the rate at which this occurs rapidly accelerates during the third decade of life. Using the hallmarks of aging first described in 2013 (https://www.cell.com/fulltext/S0092-8674(13)00645-4) as their basis, Wu and colleagues catalog symptoms associated with ovarian aging in addition to potential mechanisms driving observed changes. Moreover, since the age at first pregnancy has significantly increased among reproductively competent females due to a change in societal norms, there is a growing interest in maintaining ovarian function for longer. Consequently, several strategies to delay and/or slow the rate of ovarian aging are proposed by the authors, with many drawn from long-lived animal models including dietary interventions and altered growth hormone/insulin-like growth factor-1 (GH/IGF-1) signaling. Other proposed strategies include the application of stem-cell technology and approaches that rely on the cryopreservation of reproductive material.

Declines in cognitive function with increasing age are universal among mammals and it often differs between the sexes. Prior studies in humans have largely been inconclusive about the effect of age on memory impairment, however, but confounding factors were often overlooked and/or not considered during assessment. To parse out the potential influence of female reproductive factors on memory impairment, Li et al. compared post-menopausal females to age- and education matched males to assess both objective and subjective measures of memory. Consistent with the known elevation in the risk of dementia with aging in females, they found that postmenopausal women had an increased risk of memory impairment. The reproductive history of the females was also an important factor in that there were significant differences in the degree of memory impairment as a function of age at menarche and age at menopause, respectively.

Taken together, these studies demonstrate the increasing burden imposed on global healthcare systems as the proportion of elderly individuals in the population grows. There is a continued need for a better understanding of the mechanistic bases for these changes to aid with developing new and novel interventions to slow the rate of change with aging, or to stave them off altogether.

## Author contributions

The author confirms being the sole contributor of this work and has approved it for publication.

